# Integration of Data and Information Systems Into the Health Data Strategy

**DOI:** 10.2196/70066

**Published:** 2025-10-06

**Authors:** Martin Komenda, Jakub Gregor, Daniel Klimeš, Tomáš Pavlík, Milan Blaha, Vladimíra Těšitelová, Ondřej Májek, Ondřej Ngo, Renata Chloupková, Karel Hejduk, Lenka Šnajdrová, Jiří Jarkovský, Jan Mužík, Petra Růžičková, Vlastimil Válek, Ladislav Dušek

**Affiliations:** 1 Institute of Biostatistics and Analyses, Masaryk University, Faculty of Medicine, Brno, Czech Republic; 2 Institute of Health Information and Statistics, Palackého náměstí 4, P.O.BOX 60, Prague, 128 00, Czech Republic, 420 725174940; 3 Ministry of Health of the Czech Republic, Prague, Czech Republic; 4 Department of Radiology and Nuclear Medicine, Masaryk University, Faculty of Medicine, , Brno, Czech Republic

**Keywords:** information system, health data strategy, data-driven decision support, data interoperability

## Abstract

Integrating data and information systems into national health strategies is crucial in addressing the growing health care demands. This increase is driven by an aging population and the rising prevalence of chronic diseases. Such systems enable the collection, analysis, and publication of health data and provide critical insights based on data-driven decision-making that support policy decisions, health interventions, and service delivery. Moreover, the systems enhance the capacity for public health surveillance and enable health authorities to monitor health trends, predict disease outbreaks, and effectively manage health crises such as the recent COVID-19 pandemic. This paper highlights the key aspects and characteristics that, according to international references, a well-functioning health information system and data-driven decision-making at the national level should have. We present the outputs in the form of the National Health Data-Sharing Strategy for the Czech Republic, along with successfully implemented case studies across selected domains of its health care system. The Czech National Health Information System has been established as the backbone for centralizing health data. It is a nationwide public administration tool that collects and processes data from the essential registries of public administration bodies, ministries, health services providers, or other persons submitting data to this system. It is the foundation for shaping a health care system that is responsive to patient needs, ensures efficient resource use, and promotes a patient-centered approach. Two examples are given of the tracking of fictitious patient pathways through the health care system. The take-home message of the study is a policy-oriented endorsement of comprehensive, secure, and interoperable health information systems as the basic infrastructure for modern, patient-centered, and data-driven health care. The paper strongly advocates the National Health Information System of the Czech Republic as the primary health database for designing, implementing, and governing such a system in alignment with European and global standards.

## Introduction

Across Europe, health systems are under increasing pressure to deliver sustainable, efficient, and patient-centered care. The integration of health data and digital infrastructure into national strategies is essential to meet these demands and deliver on European Union (EU) priorities, including the European Health Data Space (EHDS). With its National Health Information System (NHIS), the Czech Republic offers a mature, coherent, and actionable model based on interoperability, standardization, and public trust.

In the context of EU member states’ efforts to modernize their health care systems, the Czech NHIS offers a transferable framework for building resilient data ecosystems that citizens trust. This ecosystem covers all segments of the Czech health care system; is structured; and, thanks to its legal alignment with EU standards, is particularly relevant for countries seeking harmonization with new EU regulations such as the General Data Protection Regulation, the Digital Services Act, and the Digital Markets Act.

Robust health information systems (HISs) such as the NHIS enable real-time data sharing, support evidence-based policy decisions, and improve public health surveillance—capabilities that have proven critical during the COVID-19 pandemic. Furthermore, the Czech system’s integration supports goals such as strengthening preventive care, reducing regional inequalities, and improving research capacities. Through the use of advanced data analytics and standardized health data exchange, the Czech NHIS is an example of how national systems can evolve toward more proactive, personalized, and efficient health care, contributing to the European Commission’s overarching goal for 2019‐2025 of creating a unified EHDS. On the basis of extensive surveys conducted in 2023, it is urgent to develop the EHDS framework in consultation with stakeholders. The use of health data scenarios to promote dialogue on technical, legal, and social aspects is crucial in this regard. Using the health data scenarios to establish a good communication channel for discussing the related technical, social, and legal aspects of the EHDS is a crucial step in this process [1].

This viewpoint highlights the Czech Republic’s policy-oriented approach to health data strategy and offers practical guidance for EU member states seeking to build modern, data-driven health care systems. The Czech model emphasizes data protection, transparency, and public trust. It calls for legal alignment with EU frameworks, cooperation between different stakeholders, and long-term investment in data literacy and sustainability. Positioned as a case study, it highlights the key elements of effective national HISs—governance, methodology, and technology—while aligning with broader European and global trends. It advocates for a structured yet flexible data infrastructure as the foundation for better health outcomes and public health resilience.

## State-of-the-Art Systems and Strategies

### Health Information Systems

HISs vary globally in design and capability, evolving in response to technological advances and the increasing role of data in public health. Effective HIS design requires understanding user needs; ensuring data security; and building flexible, scalable systems. A notable approach is the 4-stage ontology-based method, which uses participatory design to capture user knowledge, develop and implement ontologies, and test usability to ensure system effectiveness [2]. HIS design must take into account diverse health care subdomains (eg, hospitals and primary care centers) and stakeholders, ranging from patients to laboratory technicians. National systems often operate as uncoordinated vertical programs, highlighting the need for unified standards [3]. Feature modeling helps map system functionalities, while reference architecture guides domain scoping, modeling, and design through tools such as context and layered diagrams to ensure system-wide coherence and usability across subdomains [4].

### Health Data Strategies

Central health data and information system strategies offer several advantages. However, it is essential to balance these advantages with considerations regarding data privacy, security, and ethical use of health information. Making sure that patient data are handled with utmost confidentiality and security is crucial in implementing such systems. Several countries have implemented national strategies for data sharing or opening, recognizing the value of open data in driving innovation, transparency, and improved service delivery, including (but not only) the health care sector. These national strategies make nonsensitive data publicly available for transparency, encouraging innovation and allowing third parties to create value-added services. Some notable examples of organizational, methodological, and technological backgrounds in selected experienced countries are shown in Multimedia Appendix 1 [5-34]. Each mentioned country and region uses data strategies and HISs carefully designed to improve health care delivery, policymaking, and public access to information.

## Governance, Methodology, and Infrastructure

### Data System

Regarding health data in the Czech Republic, several bodies play a crucial role in the organizational, methodological, and technological backgrounds. These bodies form a robust system based on tangible outputs by being interconnected and sharing the fundamental idea of opening up the NHIS, which can be followed by other EU member states. The close and intensive collaboration with the Digital and Information Agency (DIA), as a leading authority responsible for the digitization of public administration, is an essential support; this agency brings a national perspective and manages the processing and publication of data. Multimedia Appendix 2 documents the current status in the Czech Republic, divided into different areas needed to coordinate the health data agenda sustainably.

### Organizations and Initiatives

The Ministry of Health of the Czech Republic [35] oversees health data legislation, manages departmental agendas, and sets priorities for analytical tasks in health policy. The Institute of Health Information and Statistics [36] plays a central role in collecting, analyzing, and presenting health data, while also supporting policymaking, public information, and international cooperation. The DIA [37] supports digital services; data management; standardization; and innovation across public administration, including health care. The Czech Medical Association of Jan Evangelista Purkyně [38] contributes to physician education and acts as a domain guarantor, ensuring accurate interpretation of published NHIS data.

### Methodological Frameworks

The methodological background determines the direction of the development of the data agenda in the Czech health care sector. The Strategic Analyses of Health Sector Needs [39] identify key challenges in Czech health care, such as aging populations, rising costs, and workforce shortages. They propose measures to improve access, quality, prevention, infrastructure, digitization, and legislation, while promoting patient empowerment and public health. The National Health Data-Sharing Strategy [40] outlines legal, methodological, and technical frameworks for secure and effective health data sharing across institutions, ensuring protection of personal data and supporting research and health care improvement. It defines levels of data access, from open datasets to controlled research use.

### IT Tools

The Czech NHIS [41] is a central platform that integrates and manages health care data, which enables information sharing among stakeholders and supports health policy, service planning, and public health monitoring with reliable and timely data. NHIS has adopted the following crucial attributes based on the Czech stakeholders’ and involved teams’ long-term multidisciplinary experience. The National Health Information Portal (NHIP; Multimedia Appendix 3) [42] serves as a central gateway in Czech health care, offering the public, professionals, and institutions reliable, up-to-date information on health, prevention, treatments, and patients’ rights. It ensures easy access to essential content and helps users navigate the health care system effectively [43].

### Digital Transformation and Data Standardization in eHealth in the Czech Republic

The digital transformation of health care, with NHIS as a core component, depends on broader national developments. The health sector builds on these national structures, extending them with health care–specific attributes and services. Health care providers generate most health data during routine care, while health insurance companies centralize these data for reimbursement. Centralized data collections, especially NHIS, go beyond routine data by adding clinical, standardization, and analytical value, enabling long-term evaluation of health care effectiveness in the Czech Republic.

**Figure 1.**
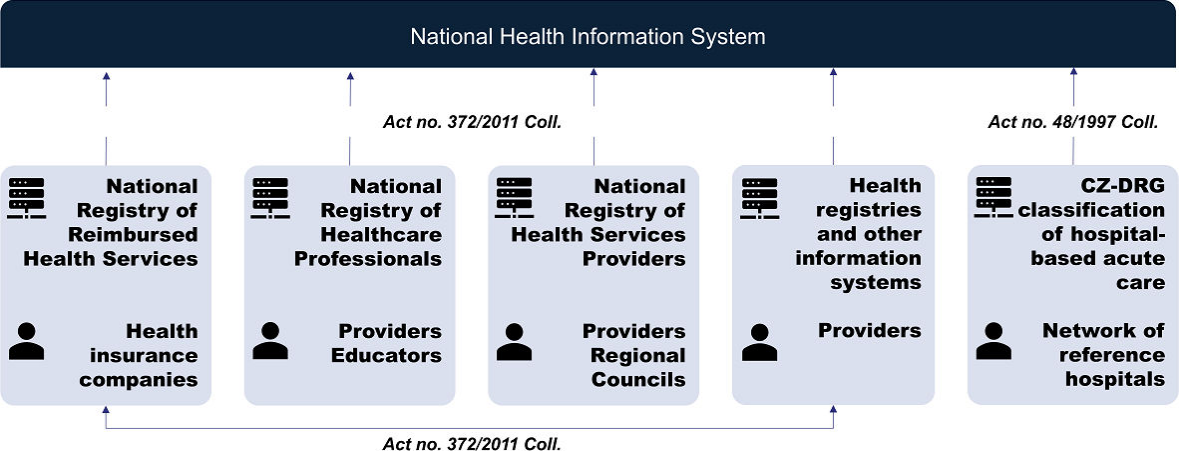
The basic components and data providers of National Health Information System (NHIS). CZ-DRG: Czech diagnosis-related group.

Primary health care data in the Czech Republic are recorded in various provider systems, which currently lack uniform standards, although all must meet basic legal requirements. Despite the coexistence of old or obsolete and modern information systems, reporting to health insurance companies follows a structured framework. Ongoing reforms, including the EHDS and the national digitization strategy, aim to introduce standardized discharge summaries, imaging, and laboratory reports. These efforts represent a shift toward more unified and quality-assured data management. Figure 1 simplistically shows the origin of the data from the perspective of the basic NHIS components and the entities responsible for entering the data, including their quality.

## National Implementation in the Czech Republic

### Overview

The NHIS is being developed as the backbone for centralizing health data (Multimedia Appendix 4). It is a nationwide public administration tool that collects and processes data from the essential registries of public administration bodies, ministries, health services providers, or other persons submitting data to this system. NHIS and its infrastructure could serve as an inspiration and potential model for other EU member states. The conditions of management and access to these data are governed by the legislation in force. The following components are essential to the NHIS part:

- National Health Registries (National Registry of Hospitalized Patients, National Cancer Registry, National Diabetes Registry, and others)
- National Registry of Reimbursed Health Services and the Czech diagnosis-related group classification system and its reference database
- National Registry of Health Services Providers and National Registry of Healthcare Professionals
- Data from death certificates
- Data from statistical surveys (ie, European Health Interview Survey and European Health Examination Survey)

### Patient Pathway Monitoring Through the Health Care System

The following section provides a brief description of a fictitious patient’s pathway [44] illustrating how the Czech health care system operates using centrally controlled data recording in the NHIS. The motivation is to describe how these patients go through the various examinations and where corresponding administrative data are recorded.

### Colorectal Cancer

A man was diagnosed with colorectal cancer during colorectal cancer screening; more specifically, a malignant neoplasm of the rectum, that is, the C20 diagnosis according to the International Classification of Diseases, 10th Revision. The management of colorectal cancer involves a structured, multidisciplinary approach that follows established clinical pathways, which is briefly described as follows:

1. After the diagnosis of colorectal cancer was confirmed, the patient was referred to specialists (gastroenterologists, oncologists, and surgeons). They conducted further examinations to determine the stage of the disease and suggested the optimal course of treatment.
2. Treatment always depended on the stage and location of the tumor. It might involve surgery, chemotherapy, targeted therapy, immunotherapy, radiotherapy, or a combination of these. Treatment took place in accredited health care facilities specialized in cancer care.
3. Upon the completion of primary treatment, the patient attended regular checkups to detect any recurrence or complications early. This follow-up included regular checkups with specialists and additional examinations if necessary.

**Figure 2.**
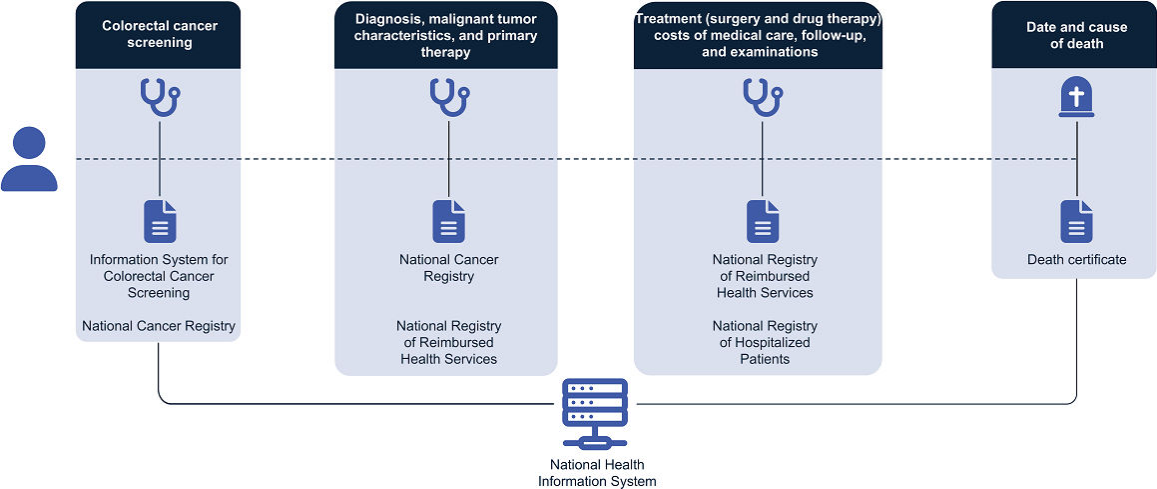
A pathway of a patient with cancer through the health care system.

The recording of information related to the disease and its treatment in the NHIS (Figure 2) is as follows:

- The Information System for Colorectal Cancer Screening collects data on the screening tests performed, their results, and subsequent diagnostic and therapeutic procedures.
- The National Cancer Registry contains information on all newly diagnosed cancers and includes demographic information about each patient and details of diagnosis, treatment, and outcomes.
- The National Registry of Reimbursed Health Services records information on health care reimbursed through public health insurance (ie, information on the health services provided during the diagnostic process and treatment, medication, the cost of treatment, or data on the health services provider).
- The death certificate sets out basic information about a person’s death, such as identification of the deceased, cause of death, any suspected unusual circumstances, and medical examination.

**Figure 3.**
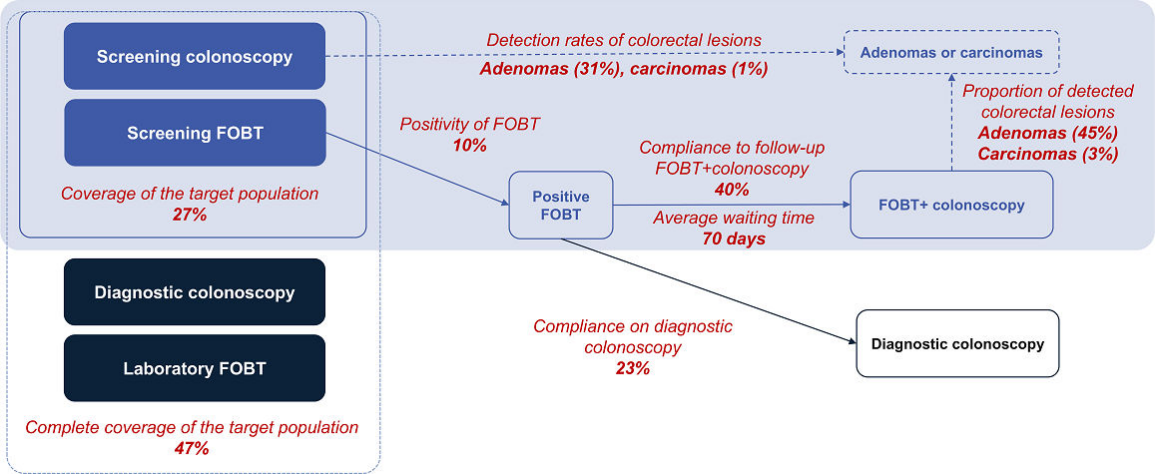
Results of key performance indicators (colonoscopy and fecal occult blood test [FOBT] coverage, FOBT positivity, adherence to colonoscopy recommendations, detection of findings) for colorectal cancer screening at the population level in the Czech Republic in 2021.

Colorectal cancer screening, introduced in the Czech Republic in 2000, is vital in reducing the population burden [45,46]. Evaluation of the National Colorectal Cancer Screening Program, based on available NHIS data, covers patient trajectories and performance indicators. Figure 3 shows the key performance indicators of the colorectal cancer screening program in the Czech Republic for asymptomatic men and women aged ≥50 years at the population level in 2021. Performance indicators are estimated from several data sources. The target population for the program in 2021 was approximately 4.1 million people. In the same year, around 44,000 preventive colonoscopies were performed (13,000 screening colonoscopies and 31,000 FOBT+ colonoscopies). Other indicators include the positivity rate of the fecal immunochemical test (10%), the average waiting time for colonoscopy (70 d), and detection rates of adenomas (31%‐45%) and carcinomas (1%‐3%). The figure also shows that a certain proportion of the Czech population undergoes other examinations related to the early detection of colorectal lesions, with complete coverage (including both screening and diagnostic examinations) reaching 47% (4.1 million people). These performance indicators for colorectal screening demonstrate the positive impact of the screening program on the population burden. However, it is imperative to continue monitoring the program to improve its quality.

**Figure 4.**
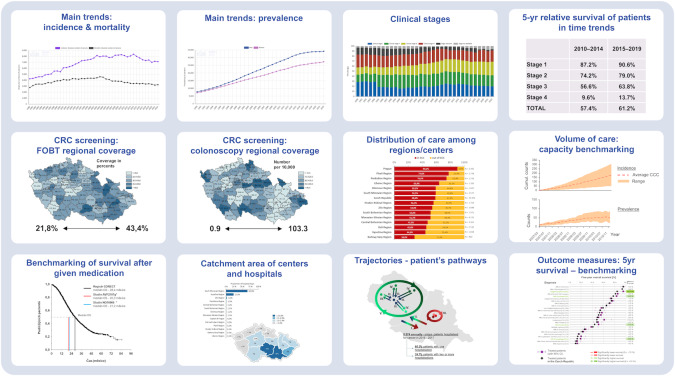
Indicators of colorectal cancer management in the Czech Republic (data source: National Cancer Registry [incidence-related parameters] and Czech Statistical Office [mortality-related parameters]). CRC: Colorectal Cancer Screening; FOBT: fecal occult blood test.

The monitoring and evaluation system of colorectal cancer screening is a specific part of the national cancer information system, which aims to monitor and evaluate the entire cancer control continuum (prevention, screening, treatment, survivorship, and end-of-life care) and is, therefore, an essential component of the Czech national cancer control plan. Figure 4 (the full-resolution version is in Multimedia Appendix 5) shows the indicators that enable monitoring of the health care system and benchmarking of the involved health care providers.

### Health Data Reporting

Across the mentioned NHIS components, the NHIP team has designed and developed a new data reporting module [47]. It covers various aspects of health care, considering the target audiences for which the tailored views of health data are intended. As of July 7, 2025, a total of 364 data outputs have been published. The individual output formats with a brief description and an example are listed in Multimedia Appendix 6 [48-54]. The release of data reporting represents a significant milestone in sharing health data for the public and professionals. Different data formats are available in one place, depending on the preferences of the data users.

### Strategic Framework: Legislation, Publishing Principles, and Target Groups

#### Overview

Various comprehensive results have been developed based on two crucial documents mentioned in the Methods section: (1) the strategic analyses of health sector needs and (2) the National Health Data-Sharing Strategy, a guiding methodological pillar fully approved by the Ministry of Health of the Czech Republic. The most central themes included (1) general provisions and compliance with national and international standards, (2) content methodology of health data access, (3) institutionalization and author responsibilities, and (4) data publication and communication channels. The effective management of access to and distribution of centralized data involves numerous steps, including standardizing the primary data from NHIS; ensuring the data are collected, followed up on, and edited efficiently; and ensuring it is published with regular updates while considering user feedback. Centralizing data can impact the entire health care system, which includes tens of thousands of health care providers, their founders, health insurance companies, specialized institutes, and more. Thus, the system must be efficient, secure, user-friendly, and easy to manage.

#### Legal Background

The full implementation of the NHIS provides a database for centralized data sharing. Its components now have an explicit legislative mandate. Legislation on data collection is essential to protect patient data, ensure data security, and promote transparency in health care processes. The establishment of the NHIS is covered by a legal framework, which is essential in establishing rules for collecting, processing, and sharing health information. Sharing health data between authorized entities contributes significantly to better care coordination, improving the quality of treatment outcomes, and supporting research. Given the sensitivity of health information, compliance with legislation is essential to ensure public trust in health care systems and technology. The legal framework consists of the following list of laws:

- Act No. 372/2011 Coll., on Health Services and Conditions of Their Provision
- Act No. 325/2021 Coll., on the Digitization of Health Care
- Act No. 89/1995 Coll., on the National Statistical Service
- Act No. 285/2002 Coll., on the Donation, Procurement and Transplantations of Tissues and Organs and on Amendments to Some Acts
- Act No. 258/2000 Coll., on Protection of Public Health and Amendment to Some Related Acts.

NHIS enables a comprehensive and full-fledged analytical evaluation of health services focused on all segments of care, a long-term assessment of patient trajectories in the system, and a representative review of access to care. The already approved (although not yet published) draft regulation on the EHDS will have implications for NHIS both in primary data processing and secondary data use. In primary data management, implementing EHDS can be expected to place greater emphasis on standardizing the content of electronic documentation directly with health service providers and health insurance companies. These processes will increase the information value of data and its usability.

#### Data Publishing Principles

The NHIS dataset design, preparation, and publishing should respect the algorithm of dataset preparation described in a numbered list below, which always respects several principal rules: (1) individual natural persons must not be identifiable, (2) individual legal persons must not be identifiable unless expressly stated by the law, (3) secondary processing must lead to the pseudonymization of the dataset, (4) the purpose of the dataset publication must correspond to the NHIS purpose, and (5) the standardized process of approval and publishing must be adhered to. The essential requirement of a comprehensive process, which this approach meets, is to ensure the necessary completeness, validity, and overall data quality [45]. The dataset publication consists of six steps describing the critical preparation and implementation phases:

1. Proposal of a dataset concept in the form of a short description, which can be raised by any entity, usually a state institution, a health insurance company, a professional (medical) society, a research institution, or an academic institution.
2. The delivered concept is reviewed from the perspective of data availability, export feasibility, design, and personal data regulations.
3. After approval, a methodology for the dataset processing is proposed (data export from central registries, data preprocessing and cleaning, analytical adjustments, and validation mechanisms).
4. The dataset is generated in an open data standardized format according to the predefined scheme, including an obligatory description with metadata.
5. Reviewing and validating the dataset and its content is a mandatory procedure before publication (a manual approach is always needed, as well as technical control to follow open data standards and best practices).
6. Publication of the final dataset in the data reporting module under the Creative Commons Attribution 4.0 International license (CC-BY-4.0).

These rules allow complete control over methodological, legislative, and technical aspects significantly affecting health data preparation and sharing. At the same time, the processes set up align with the entire ministry’s strategic vision, which is based on the principles of evidence-based medicine and data-based decision-making.

#### Target Group Focus

**Figure 5.**
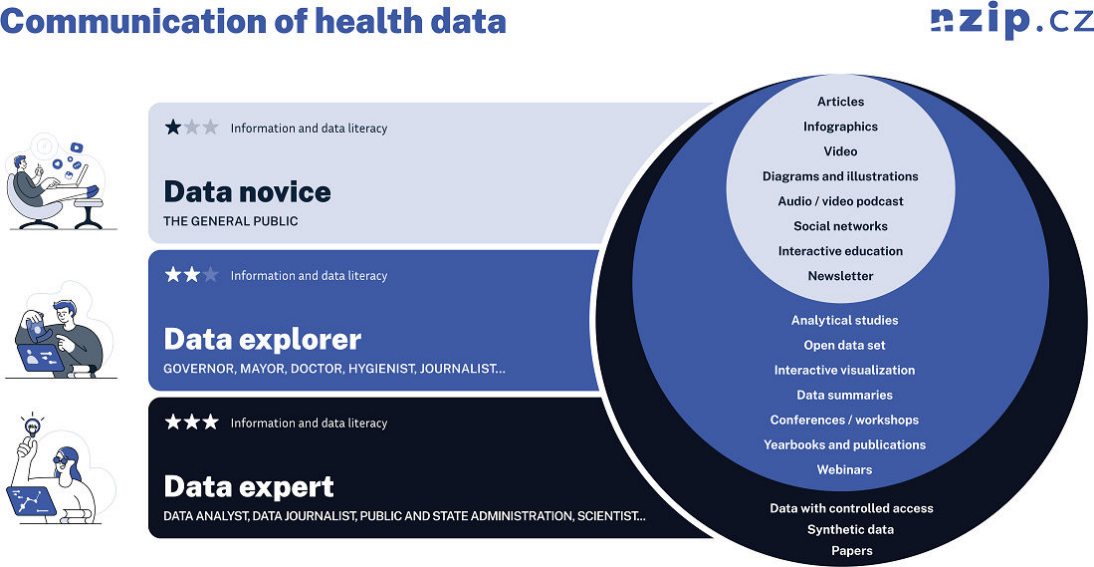
Communication target group of health data.

Effective health communication requires identifying target groups and tailoring messages to their literacy levels regarding health, information, and data. Adapting the complexity of content, language, and format not only increases understanding and engagement but also reduces the risk of misinterpretation. For instance, younger audiences may prefer digital media, while older adults may favor printed materials. Clear communication tailored to a specific audience supports informed decisions and healthier behaviors. Figure 5 illustrates the 3 main target groups according to the expected user input knowledge level, showing the different types of health data outputs.

A summary of health data use is provided in Table 1, showing the different data formats and the primary (though not exclusive) target users for each format. The motivation is to cover the largest possible group of those interested in health data and to develop health and data literacy in the Czech Republic in the long term through published data.

**Table 1.** Relationship between target user groups and published health data formats.

Output format	Data novice	Data explorer	Data expert
Open data		✓	✓
Data summaries		✓	✓
Analytic studies		✓	✓
Yearbooks and publications	✓	✓	✓
Interactive visualizations and infographics	✓	✓	✓
Dedicated analytical portals		✓	✓
Synthetic data			✓

## Discussion

### Key Lessons Learned

The Czech Republic’s National Health Data-Sharing Strategy, jointly managed by the Ministry of Health, the Institute of Health Information and Statistics, and DIA, highlights several important findings. The centralization of health data through standardized national registries, electronic health records, and real-time reporting tools significantly improves health care efficiency, patient outcomes, and interoperability between health care providers, researchers, and public health authorities. The NHIP forms the backbone of data dissemination and provides interactive visualizations, open datasets, and customized data views for different users.

Privacy and security remain critical, as patient confidentiality and General Data Protection Regulation compliance directly shape data-sharing policies. To address this, the NHIS, which encompasses 61 data agendas (including national registries, prevention programs, and statistical surveys), uses advanced encryption and anonymization techniques, as well as strict access controls that enable authorized yet secure access.

The NHIS is an example of a transformative change in health care methodologies and highlights several key elements:

- Privacy and confidentiality—the protection of patient data is crucial to prevent discrimination and promote public trust and participation in health research.
- Importance for research and public health—data stratified by sex, age, education, and ethnicity are crucial for identifying inequalities and guiding targeted public health interventions (eg, World Health Organization Health Inequality Data Repository and European Cancer Information System).
- Diverse stakeholder concerns—patients, health services providers, and policymakers have different needs and expectations that must be taken into account in the design of health data systems and the development of policy measures.
- Technological and analytical challenges—advanced technologies and sophisticated analytical methods are essential to effectively manage the complexity and volume of health data.

The digital transformation of health care in the Czech Republic, supported by the aspects mentioned in Methods (subsection: Digital Transformation and Data Standardization in eHealth in the Czech Republic), has been enhanced by new tools currently being developed and tested: a new synthetic data system (a promising innovative tool for data analysis experts to conduct sophisticated analytical studies on NHIS patient data) and artificial intelligence chatbots (which answer questions about health statistics in simple language).

### Actionable Recommendations for Other European Countries

The NHIS in the Czech Republic forms the basis for designing a health care system that responds to patients’ needs, uses resources efficiently, and pursues a patient-centered approach. When establishing such a robust framework for a specific area, such as health care, EU member states should focus on the following key areas:

- Compliance with regulatory and legal requirements—alignment with local, national, and international health care and legal regulations.
- Data security—protection of sensitive health data and implementation of encryption, user authentication, and access control, including a robust IT infrastructure.
- Data management and validation—ensuring the integrity and accuracy of health data, including automatic and semiautomatic control mechanisms that guarantee valid and correct data.
- Standardization—using health care standards (ie, International Classification of Diseases, 10th Revision or the Czech diagnosis-related group) and uniform lists of values among all related areas (ie, population and demography). A practical example is the shared medical record, which will enable secure access to patients’ preventive care and screening data across all health care providers. Its implementation is scheduled for the end of 2025. Another example of planned standardization is the early introduction of structured and standardized discharge reports, imaging reports, and laboratory results as part of the national strategy for the digitalization of health care.
- Multidisciplinary team—appropriate composition of experts with in-depth specialist knowledge (with or without the support of subcontractors), representing all necessary professional groups (data architect, data manager, analyst, developer, web designer, communication expert, language expert, and a representative of an expert medical society) and ensuring regular internal communication.
- Support for leading institutions—all leading representatives and committees of involved stakeholders must be informed and involved in all important decisions.
- Communication and public relations—focus on the actual needs of target groups and clear presentation of data outputs, including the establishment of professional communities through conferences and workshops.
- Data outputs by target groups—provision of outputs tailored to user requirements and preferences (open data, data summaries, infographics, interactive visualizations, and synthetic data).
- User-friendliness, accessibility, and experience—the design and format of the published outputs must comply with user experience or user-friendliness rules and accessibility rules (especially for people with disabilities) and graphical and user-friendly requirements.
- Technological sustainability—minimization of technological debt and continuous development of information systems and portals based on IT trends, user behavior, and needs arising from actual use.
- Financial sustainability—efficient allocation of resources, taking into account development, maintenance, and operating costs, including process optimization, to ensure long-term cost-effectiveness.

### Limitations of the Study, Especially With Regard to the Transferability of Findings

This case study examines how health data can be collected, integrated, and published at a national level. Although it is limited to one country, many of the findings are transferable to other countries, depending on the structure of the health care system, the proportion of public health insurance, and the availability of IT data. The main advantages of the Czech health care system in terms of data integration are (1) the high proportion of health services covered by public health insurance and (2) a registry in which data on these health services are centralized. This enables system-wide evaluation and analysis of diagnoses, areas of care, medications, equipment, procedures, and their reimbursement. On the other hand, there are considerable differences in the complexity of data within individual agendas and topics. Although administrative data on health services reimbursed from public health insurance cover the entire spectrum of care, procedures, and medications, clinical information is generally lacking. This is only recorded for selected care segments or diagnosis groups in specialized registries (eg, cancer care, cardiovascular surgery, and blood donation). Given the scope and nature of the data, all interpretations and conclusions drawn from NHIS data should always be made with caution and supported by sound professional knowledge and expertise, taking into account other integrated health data systems. Another significant limitation is the quality of the input data and the lack of motivation on the part of the responsible institutions to enter it correctly and completely into these systems. An example of this is the National Cancer Registry, where incomplete entry of clinical stages is often a major problem.

### Conclusions

The integration of data and information systems into the Czech Republic’s national health data strategy can transform health care by improving patient outcomes and optimizing resource use, for example, through improved disease surveillance, targeted prevention (eg, colorectal cancer screening), and more efficient health care planning based on integrated patient data. To reap these benefits, policymakers have created robust interoperable infrastructures that enable real-time decision-making and evidence-based planning. Standardization across the health sector is crucial to ensure seamless communication between stakeholders and prepare for participation in EHDS, which requires legal, technical, and methodological alignment with international frameworks. The Czech Republic’s NHIS is an important infrastructure for alignment with the EHDS. Successful integration into the EHDS requires the modernization of the NHIS so that it functions as a system compatible with European standards for the secure sharing and secondary use of health data. With its universal health care model and strong digital infrastructure, the Czech health care system is well positioned to take a leading role in regional efforts to transform digital health care. To fully exploit these advantages and set an example of sustainable data-driven health care governance in Europe, further progress is needed in the areas of infrastructure, harmonization of legislation, and data literacy and awareness among the population.

## Supplementary material

10.2196/70066 Multimedia Appendix 1 Overview of organizations and initiatives.

10.2196/70066 Multimedia Appendix 2 Crucial attributes of the National Health Information System.

10.2196/70066 Multimedia Appendix 3 Screenshot of the National Health Information Portal home page.

10.2196/70066 Multimedia Appendix 4 Overview of all National Health Information System agendas and data sources.

10.2196/70066 Multimedia Appendix 5 Indicators of colorectal cancer management in the Czech Republic (high resolution).

10.2196/70066 Multimedia Appendix 6 Overview of outputs published in the data reporting module (information valid as of July 31, 2025).

## Abbreviations

DIA: Digital and Information Agency
EHDS: European Health Data Space
EU: European Union
HIS: health information system
NHIP: National Health Information Portal
NHIS: National Health Information System
