# Supplementary material for: Integration of Data and Information Systems Into the Health Data Strategy
Source: JMIR Med Inform. 2025 Oct 6;13:e70066. doi: 10.2196/70066 (PMC12500401; doi:10.2196/70066)
Supplement: Multimedia Appendix 1 [file medinform-v13-e70066-s001.docx]

Multimedia Appendix 1 Overview of organisations and initiatives, methodological frameworks and IT tools in selected developed countries

|  | **Organisations and initiatives** | **Methodological frameworks** | **IT tools** |
| --- | --- | --- | --- |
| EU | eHealth Network [5]  European Centre for Disease Prevention and Control [6] | European Data Strategy [7]  European Health Data Space [8] | European Health Information Gateway [9] |
| USA | U.S Centres for Disease Control and Prevention [10]  National Center for Health Statistics [11]  Office of the National Coordinator for Health Information Technology [12] | Federal Health IT Strategic Plan [13]  National Action Plan to Improve Health Literacy [14] | National Health and Nutrition Examination Survey [15]  National Healthcare Surveys [16] |
| UK | National Health Service [17]  General Practice Data for Planning and Research [18] | NHS Long Term Plan [19]  Digital Health Laws and Regulations [20] | NHS Federated Data Platform [21] |
| Canada | Canada Health Infoway [22]  Canadian Institute for Health Information [23] | Pan-Canadian Health Data Strategy [24]  Pan-Canadian Health Data Content Framework [25] | Open Government portal [26] |
| Australia | Australian Digital Health Agency [27]  Australian Institute of Health and Welfare [28]  Australian Bureau of Statistics [29] | National Digital Health Strategy [30]  Strategic Directions 2022–2026 [31] | My Health Record [32]  The Healthcare Identifiers Service [33]  Health Data Portal [34] |
