# Supplementary material for: Integration of Data and Information Systems Into the Health Data Strategy
Source: JMIR Med Inform. 2025 Oct 6;13:e70066. doi: 10.2196/70066 (PMC12500401; doi:10.2196/70066)
Supplement: Multimedia Appendix 2 [file medinform-v13-e70066-s002.docx]

Multimedia Appendix 2 Crucial attributes of the National Health Information System

| **Attribute** | **Description** |
| --- | --- |
| Comprehensive data collection | The HIS should be able to collect a wide range of health data, including patient demographics, clinical data, and healthcare service utilisation. These data must be comprehensive to support various health system functions. |
| Quality data management | The system must manage data efficiently, ensuring accuracy, completeness and reliability. Data quality is critical for making sound health decisions and policies. |
| Timely data access and publication | A robust HIS provides timely access to health data, enabling healthcare professionals and policymakers to make informed decisions quickly. |
| Interoperability | The ability of the HIS to work with other systems and exchange data effectively is crucial. Interoperability ensures that different health information systems can communicate and exchange data efficiently. |
| User-centered design | The HIS should be designed with the end user in mind, ensuring ease of use and relevance to healthcare providers’ and patients' needs. |
| Scalability and adaptability | The system should adapt to changing healthcare needs and technologies and be scalable to accommodate data volume and complexity growth. |
| Security and privacy | It must ensure the confidentiality and security of patient data, complying with legal standards and ethical considerations. |
| Support for decision-making and policy formation | The system should aid in generating information that enables decision-makers at all health system levels to identify problems, make evidence-based decisions and allocate resources optimally. |
| Integration with other data sources | It should integrate with different data sources, such as health facilities, population-based surveys, civil registration and vital statistics systems, to meet diverse informational needs. |
