# Supplementary figures and images for: Integration of Data and Information Systems Into the Health Data Strategy
Source: JMIR Med Inform. 2025 Oct 6;13:e70066. doi: 10.2196/70066 (PMC12500401; doi:10.2196/70066)

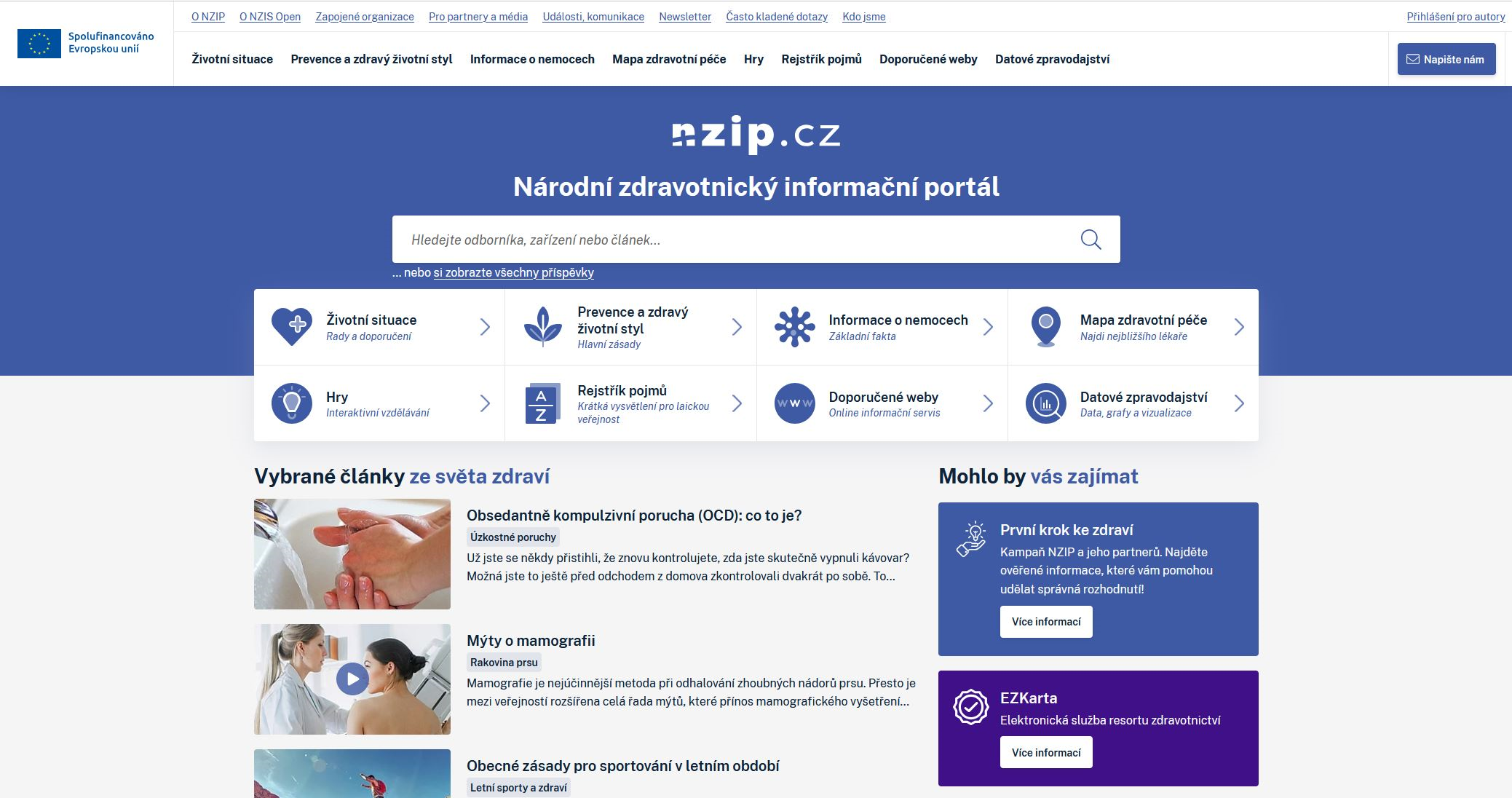

Supplement: Multimedia Appendix 3 [file medinform-v13-e70066-s003.png]

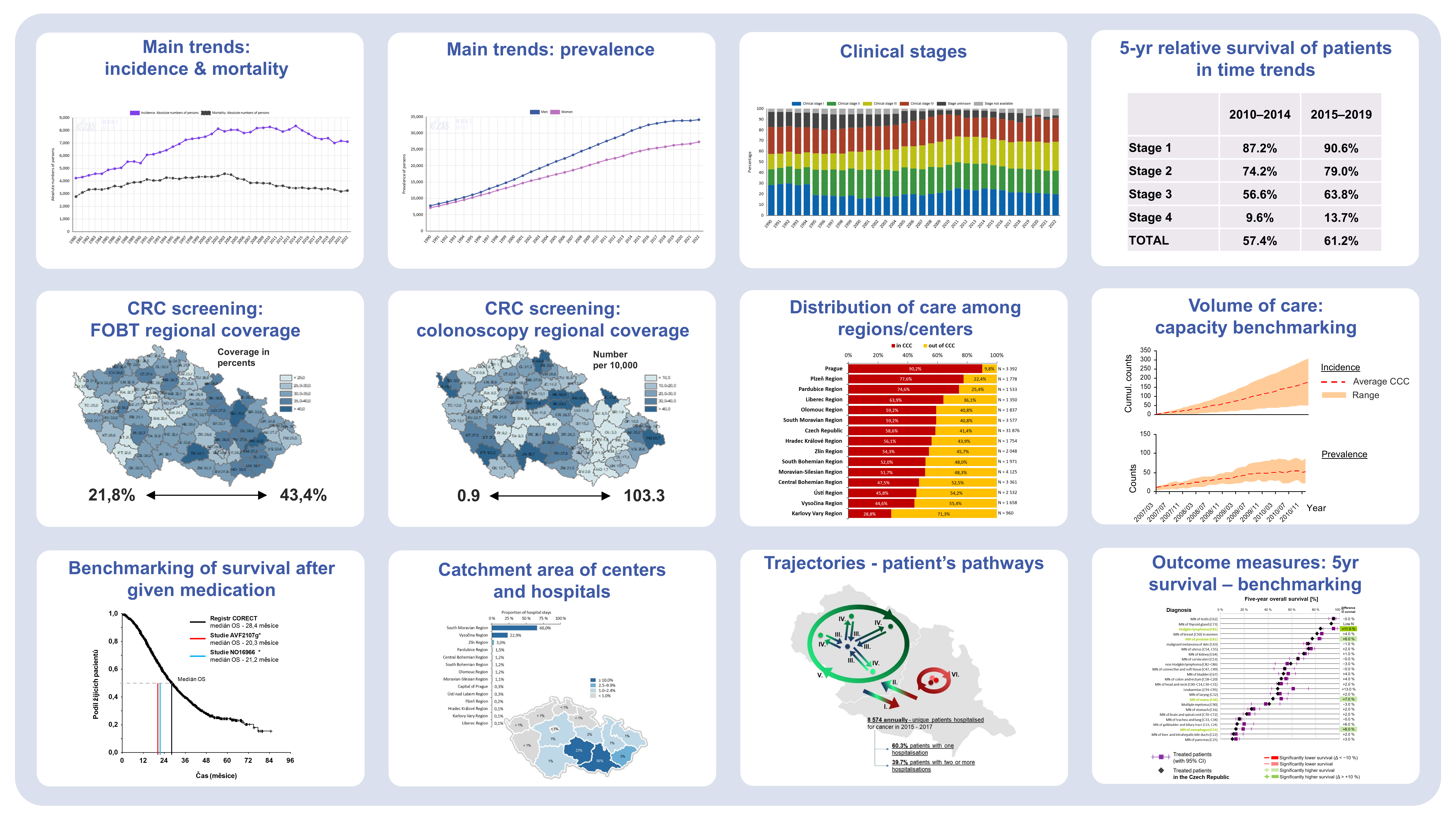

Supplement: Multimedia Appendix 5 [file medinform-v13-e70066-s005.png]
