# Supplementary material for: Integration of Data and Information Systems Into the Health Data Strategy
Source: JMIR Med Inform. 2025 Oct 6;13:e70066. doi: 10.2196/70066 (PMC12500401; doi:10.2196/70066)
Supplement: Multimedia Appendix 4 [file medinform-v13-e70066-s004.docx]

Multimedia Appendix 3 Overview of all NHIS agendas and data sources

| **National registries and information systems** | | | **Prevention and screening programmes** | **Statistical surveys** |
| --- | --- | --- | --- | --- |
| Central Vaccination Registry | CZ-DRG Classification System | National Registry of Drug Addict Therapy | Central Registry of Data from Population-Based Prevention Programmes | Information about Health Services Provider |
| Lists of values | Death Certificate Information System: Death Records Database | National Registry of Occupational Diseases | Information System for Laboratory Newborn Screening | Number of Beds |
| Data Support for Negotiated Procedures | National Diabetes Registry | National Registry of Autopsy and Toxicological Examination Performed at Forensic Medicine Departments | Information System for Cervical Cancer Screening | Wages and Salaries in the Healthcare System |
| Control Centre for Intensive Care | National Information System on Palliative Care | National Registry of Health Services Providers | Information System for Lung Cancer Screening | Annual Reporting of the Number of Adverse Events for Central Evaluation |
| Population health indicators and HSPA (Health System Performance Assessment) | National Information System on Mental Health Care | National Registry of Reproductive Health | Information System for Prostate Cancer Screening | Health Interview Surveys |
| Information System of Infectious Diseases (apart from COVID-19) | National Cardiology Information System | National Registry of Injuries | Information System for Breast Cancer Screening | Reports on Pharmacy Care |
| Information System of Infectious Diseases: COVID-19 (including vaccinations) | National Cancer Registry | National Registry of Healthcare Professionals | Information System for Colorectal Cancer Screening | Reports on Healthcare |
| Information System of Places of Health Services | National Registry of Hospitalised Patients | Portal of Medical Indicators | Information System for Newborn Hearing Screening | Healthcare: Foreign Nationals |
| Information System for the Support of Regional Concepts of Healthcare | National Registry of Reimbursed Health Services | Job Classification Registry |  | Medical Equipment |
| Information System for the Evaluation of Centres Providing Highly Specialised Care | National Registry of Intensive Care | Registry of Sexually Transmitted Diseases |  |  |
| Information System for the Evaluation of Social and Health Care | National Registry of Cardiovascular Surgery and Interventions | Tuberculosis Registry |  |  |
| Information System for Predicting Costs and Needs of Highly Specialised Care | National Registry of Joint Replacement |  |  |  |
