# Supplementary material for: Integration of Data and Information Systems Into the Health Data Strategy
Source: JMIR Med Inform. 2025 Oct 6;13:e70066. doi: 10.2196/70066 (PMC12500401; doi:10.2196/70066)
Supplement: Multimedia Appendix 6 [file medinform-v13-e70066-s006.docx]

Multimedia Appendix 4 Overview of available outputs published at Data reporting (information valid on 31 July 2025)

| **Output format** | **Description** | **Example** |
| --- | --- | --- |
| Open data  (142 in total) | A machine-readable file (typically in CSV format) which must meet several criteria under current legislation [48]. | Follow-up care coverage in psychiatry [49] |
| Data summaries  (146 in total) | A file with aggregated data (typically in XLSX format) on various aspects of healthcare such as healthcare utilisation, data on the performance of healthcare facilities, demographic distribution of patients, information on the cost of health services, etc. | Cardiac surgery performed in the Czech Republic [50] |
| Analytic studies  (17 in total) | A static report (typically in PDF format) contains graphical and tabular summaries and medical data supplemented with correct interpretations and conclusions. | Vaccination in available NHIS data [51] |
| Yearbooks and publications  (9 in total) | A comprehensive overview (typically in PDF format) of the population's health status, the use of health services and other relevant indicators. At the same time, they enable analysis of trends in disease incidence, health system efficiency and changes in health indicators over time. | Assisted Reproduction (NRHR Yearbooks) [52] |
| Interactive visualisations and infographics  (49 in total) | A modern way of graphically displaying data (business intelligence tool output or infographics) where filters can refine the view (e.g. selecting gender, region, diagnosis, etc.). | Adverse events: a basic overview over time [53] |
| Dedicated analytical portals  (8 in total) | A stand-alone web platform focused on one specific domain of Czech healthcare, where detailed reports are available in interactive graphs and tables, often supplemented by static reports. | Portal of Cancer Epidemiology in the Czech Republic [54] |
